# Clinical and Epidemiological Profile of Patients Presenting With Acute Abdomen to the Emergency Department of a Tertiary Care Hospital

**DOI:** 10.7759/cureus.67017

**Published:** 2024-08-16

**Authors:** Varsha Shinde, Yash Dixit, Pranay Penmetsa, Karthik R Nair

**Affiliations:** 1 Emergency Medicine, Dr. D. Y. Patil Medical College, Hospital and Research Center, Dr. D. Y. Patil Vidyapeeth (Deemed to be University), Pune, IND

**Keywords:** epidemiology and biostatistics, clinical profile of acute abdomen, pain management, surgical abdomen, acute abdomen

## Abstract

Background: Acute abdomen is a common and urgent clinical condition requiring prompt diagnosis and treatment. This study determines the clinical and epidemiological profile of patients presenting with acute abdomen at a tertiary care hospital.

Objective: To describe the demographic characteristics, provisional diagnoses, treatment modalities, and pain management effectiveness in patients with acute abdomen.

Methods: This prospective observational study was conducted in the Emergency Medicine department at Dr. D. Y. Patil Medical College, Hospital & Research Center, Pimpri, Pune, over a specified period. The study included patients presenting with acute abdomen, aged above 14 years, excluding those with traumatic acute abdomen and pregnant patients. A sample size of 146 was calculated based on the proportion of ureteric colic cases, with a 95% confidence interval and a 6% margin of error; however, a total of 176 patients were included in the study. Data collection involved recording demographic details, clinical features, provisional diagnoses, and pain scores, as well as performing required blood investigations and ultrasonography. Pain scores were assessed before and after treatment. Patients will be given non-steroidal anti-inflammatory drugs (NSAIDs) or opioid analgesia, depending on the clinical severity. Emergency medicine residents, in consultation with the on-call consultant, determined the disposition of patients, deciding if they required surgical or conservative management.

Results: The study found that the majority of patients, n = 130 (73.86%), were aged 26-50 years, with cases n = 103 (58.52%) being males and cases n = 73 (41.48%) females. Acute appendicitis was the most common diagnosis, n = 41 (24.43%), followed by urolithiasis n = 33 (18.75%). Surgical interventions were required for n = 78 (45.08%) of patients, highlighting the urgent nature of these conditions. Pain management was effective, with significant reductions in pain scores post-treatment (mean visual analog score (VAS) decreased from 6.22 to 2.33, and mean numerical rating score (NRS) from 6.05 to 2.10; p < 0.001).

Conclusion: The study underscores the high prevalence of gastrointestinal and renal conditions in patients with acute abdomen, particularly in middle-aged adults. The high rate of surgical interventions reflects the urgent nature of these conditions. Significant reductions in pain scores demonstrated effective pain management. Comprehensive care strategies are essential for optimizing patient outcomes. Future research with larger sample sizes and multi-center participation is recommended to validate these findings and enhance management protocols for acute abdomen.

## Introduction

The term "acute abdomen" denotes a wide spectrum of surgical, medical, and gynecological conditions ranging from benign to life-threatening, necessitating hospital admission, thorough investigations, and prompt treatment [1]. It encompasses a diverse array of potential diagnoses, spanning from self-limiting to critical conditions [2]. Abdominal pain constitutes a primary cause for emergency room visits, with the majority of cases being benign and transient, although a subset necessitates urgent attention due to serious intra-abdominal pathology [3].

An acute abdomen denotes the abrupt manifestation of intense abdominal pain, typically linked with exigent medical intervention due to underlying pathologies. These pathologies span across gastrointestinal, genitourinary, vascular, and obstetric/gynecologic systems. Common indicators encompass severe abdominal pain and vomiting, along with prevalent clinical signs such as tenderness and guarding [4]. Prominent causes of acute abdomen comprise acute appendicitis, cholecystitis, pancreatitis, and diverticulitis, each characterized by distinct inflammatory or infectious processes affecting the respective organs [5]. Additionally, acute peritonitis, precipitated by hollow viscus rupture or secondary to inflammatory bowel disease or malignancy, constitutes a critical etiology demanding prompt intervention [6]. Vascular emergencies such as mesenteric ischemia and ruptured abdominal aortic aneurysm represent life-threatening conditions contributing to acute abdomen through compromised blood supply to abdominal organs or catastrophic vessel rupture [7]. Urologic conditions, including ureteral colic and pyelonephritis, may manifest with acute abdominal pain, warranting thorough assessment for timely diagnosis and treatment [8]. Moreover, small bowel obstruction, often attributed to mechanical causes, can precipitate acute abdomen and necessitate urgent intervention [9,10].

Various physicians, manage patients with abdominal pain in the emergency department (ED), a challenging task often linked to higher readmission rates. Optimal therapy begins with careful optimization of the patient's clinical status. The initial assessment includes a comprehensive medical history, physical examination, and appropriate use of laboratory tests and imaging studies [7]. Pain location is crucial for diagnosis, as localized pain suggests a specific issue, while diffuse pain with free air may indicate peritonitis, characterized by absent bowel sounds, rebound tenderness, and guarding on palpation [11].

Abdominal pain is a common reason for visits to the emergency department (ED), with its prevalence estimated to be between 7% and 11%, according to different studies [12]. Acute diverticulitis is a notable concern, particularly affecting men and primarily impacting the left colon, as highlighted in a study from Saudi Arabia [13]. Healthcare delivery faces challenges, as Sri-on et al. reported that 50% of readmission cases involved misdiagnosis, delayed treatment, and inappropriate discharge advice, pointing to potential flaws in initial evaluations and management strategies [14]. Frequent readmissions to EDs within short periods can indicate insufficient care, leading to complications, increased mortality rates, higher costs, and ED overcrowding [15].

This study estimates the proportion of surgical abdomen to medical emergency and their disposition, presentation of the surgical abdomen in the emergency department and clinical profile. This study estimates the difference in pain scores before and after emergency management. Addressing these challenges is essential not only for enhancing patient outcomes and healthcare quality but also for alleviating the strain on healthcare resources and optimizing emergency department efficiency. Additionally, the limited availability of data on this topic, particularly within the Pune, Maharashtra region, emphasizes the pioneering nature of our study. While studies have explored challenges in healthcare delivery related to acute abdominal conditions, the specific context of Pune, Maharashtra, remains largely unexplored. By embarking on this research endeavor, the present study aims to fill this notable gap in the literature, providing novel insights into the clinical and epidemiological profile of acute abdomen presentations in our local healthcare setting. This pioneering effort not only contributes to advancing scientific knowledge but also lays the groundwork for future research initiatives and targeted interventions aimed at improving healthcare outcomes for patients presenting with acute abdomen in Pune, Maharashtra.

## Materials and methods

Study design

The study was designed as a prospective observational study conducted on patients presenting with acute abdomen to the Emergency Medicine department at Dr. D. Y. Patil Medical College, Hospital & Research Center, Pimpri, Pune.

Period of study

The study was conducted from November 2022 to July 2023.

Sample size calculation

The sample size was calculated based on the proportion of ureteric colic among cases presented to the Emergency Medicine department, as reported by Chanana et al. (2015). With an acceptable difference of 6% and a confidence interval of 95%, the sample size was determined to be 146 using WinPepi version 11.38 (J. H. Abramson, Brixton Health, United Kingdom).

**Figure 1:**
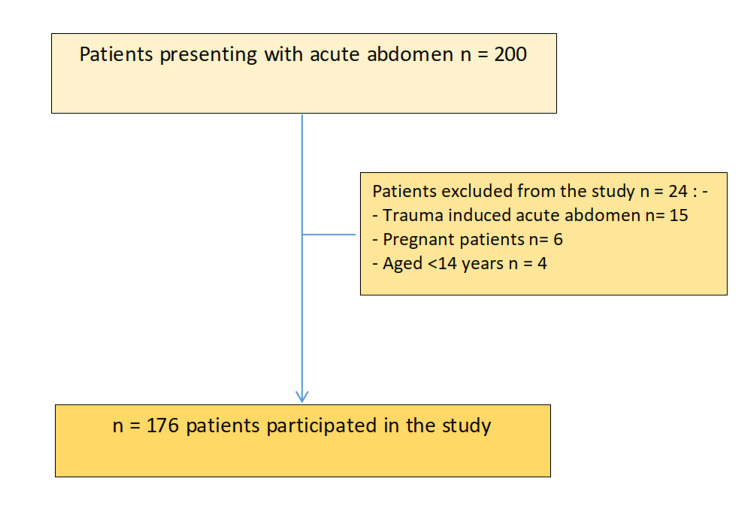
Flow diagram showing patient selection.

The formula used for calculating sample size for estimating a proportion is: n = Z^2 ^× p × (1−p)​/E^2^,

where: n = required sample size, Z = Z-score corresponding to the desired level of confidence, p = estimated proportion or prevalence of the event in the population, and E = margin of error (acceptable difference). Figure 1 shows the flow diagram of patient selection.

Inclusion criteria

Patients presenting with clinical features of acute abdomen were included and only individuals aged above 14 years were considered eligible for participation.

Exclusion criteria

Cases of traumatic acute abdomen were excluded, and pregnant patients were also excluded from the study.

Study protocol

The study protocol began with the acquisition of written and informed consent from eligible patients, ensuring adherence to ethical standards. Proforma was filled for the patients included in the study with their clinical profile and epidemiology after obtaining consent for the study (see Appendix, Table 7). For pain management, patients will be given non-steroidal anti-inflammatory drugs (NSAIDs) or opioid analgesics, depending on the clinical severity. Pain scores were recorded before and after treatment in an Excel sheet (Microsoft Corporation, Redmond, Washington, USA). Data on patients transferred to the operating theater (OT), those who received active resuscitation, and those requiring conservative management were also documented.

Patients who met the predefined inclusion criteria were enrolled consecutively to form a representative study cohort. Data collection was followed by systematic documentation of the required information. The collected data were entered into Excel spreadsheets for subsequent analysis. Statistical analysis with paired t-test was done to analyze the clinical and epidemiological profiles of patients presenting with acute abdomen in the Emergency Department of the tertiary care hospital, enhancing the understanding of the studied phenomenon.

## Results

Table 1 provides a distribution of cases across various age groups, showing the number of cases and their corresponding percentages. The age group 26-50 years has the highest number of cases, with 130 cases accounting for 73.86% of the total. This is followed by the 14-25 years age group, which has 32 cases, representing 18.18%. The age group 51-75 years has 12 cases, making up 6.82% of the total, while those older than 75 years have the fewest cases, with just two cases, constituting 1.14%. Overall, the total number of cases is 176, distributed across these age groups.

**Table 1:** Distribution of age group.

Age group	Cases (n)	Percentage
14-25	32	18.18%
26-50	130	73.86%
51-75	12	6.82%
>75	2	1.14%
Total	176	100.00%

Table 2 illustrates the distribution of cases by gender, detailing both the number of cases and their respective percentages. Out of a total of 176 cases, 73 are female, which constitutes 41.48% of the total. In comparison, there are 103 male cases, making up 58.52% of the total. This indicates that a higher proportion of cases are among males compared to females.

**Table 2:** Gender distribution.

Sex	Cases (n)	Percentage
Female	73	41.48%
Male	103	58.52%
Total	176	100.00%

Table 3 presents a breakdown of provisional diagnoses for a total of 176 cases. Each diagnosis is listed along with the number of cases it represents and its corresponding percentage of the total. The most prevalent conditions include acute appendicitis, with 41 cases (23.30%), and renal calculi, with 33 cases (18.75%). Other significant diagnoses include pancreatitis (12 cases, 6.81%), bowel obstruction (12 cases, 6.81%), ectopic pregnancy (eight cases, 4.54%) and cystitis (seven cases, 3.97%). Less frequent diagnoses, such as hepatitis, ectopic pregnancy, and ovarian torsion, each represent a smaller percentage of the total cases. This breakdown helps to understand the distribution and prevalence of various medical conditions within this patient population. 

**Table 3:** Provisional diagnosis.

Provisional diagnosis	Cases	Percentage (%)
Renal calculi	33	18.75
Hernia	5	2.84
Hepatitis	2	1.13
Ruptured ectopic	2	1.13
Acute appendicitis	41	23.30
Acute cholecystitis	5	2.84
Bowel obstruction	12	6.81
Bowel perforation	6	3.40
Colitis	2	1.13
Constipation	4	2.27
Cystitis	7	3.97
Decompensated liver disease	6	3.40
Ectopic pregnancy	8	4.54
Gallstones	10	5.68
Inflammatory bowel disease	2	1.13
Liver abscess	3	1.70
Ovarian cyst	5	2.84
Ovarian torsion	1	0.56
Pancreatitis	12	6.81
Peptic ulcer	6	3.40
Testicular torsion	4	2.27
Total	176	100.00%

Table 4 outlines the disposition of patients, detailing the number of cases and their corresponding percentages. Out of 176 total cases, 78 patients (44.31%), were shifted to the operating theater (OT) for surgical intervention. Meanwhile, 98 patients, representing 55.68%, were transferred to other wards for further medical treatment or observation. This distribution indicates that a majority of the patients required surgical procedures, while a significant portion needed continued care in different hospital wards.

**Table 4:** Disposition of patients.

Disposition	Cases (n)	Percentage
Shifted to OT	78	44.31%
sifted to other wards	98	55.68%

Among the patients presenting with acute abdomen, pain scores were recorded, and the following analgesics were administered: injection of Tramadol 50 mg IV to 80 patients (45.45%), injection of Diclofenac 75 mg IM to 70 patients (39.77%), and both analgesics to 26 patients (14.77%). Table 5 compares the visual analog scores (VASs) of patients before and after treatment, indicating the effectiveness of the treatment. The mean VAS before treatment was 6.22, with a standard deviation (SD) of 2.32, suggesting moderate to severe pain levels on average with some variability among patients. After treatment, the mean VAS significantly decreased to 2.33, with an SD of 1.59, indicating much lower pain levels. A paired t-test was applied, and the p-value is less than 0.001, demonstrating that the reduction in pain scores is statistically significant. This substantial decrease in pain scores highlights the effectiveness of the treatment in alleviating the patient's pain.

**Table 5:** Comparison of visual analog score at before and after treatment. SD: Standard deviation. Paired T-test was applied for statistical analysis.

Visual analog	Mean ± SD	p-value
Score at before treatment	6.22 ± 2.32	<0.001
Score after treatment	2.33 ± 1.59	

Table 6 compares the numerical rating scores (NRSs) of patients before and after treatment, demonstrating the impact of the treatment on pain levels. Before treatment, the mean NRS was 6.05 with a standard deviation (SD) of 2.42, indicating that patients generally experienced moderate to severe pain with some variation in pain intensity. Following treatment, the mean NRS significantly dropped to 2.10, with an SD of 1.30, reflecting much lower pain levels. The paired T-test is applied, and the p-value is less than 0.001, indicating that this reduction in pain scores is statistically significant. These results underscore the effectiveness of the treatment in significantly reducing patient pain.

**Table 6:** Comparison of numerical rating score before and after treatment. SD: Standard deviation. Paired T-test was applied for statistical analysis.

Numerical rating	Mean ± SD	p-value
Score at before treatment	6.05 ± 2.42	<0.001
Score after treatment	2.10 ± 1.30	

## Discussion

The study aimed to evaluate the clinical and epidemiological profile of patients presenting with acute abdomen in the emergency department of a tertiary care hospital. The parameters assessed included age and gender distribution, provisional diagnoses, disposition of patients, and the efficacy of treatment in terms of pain reduction.

Age and gender distribution

In the present study, the majority of patients n = 130 (73.86%) fell within the 26-50 years age group. This finding aligns with the literature, which indicates that conditions like appendicitis, intestinal obstruction, cholecystitis, and diverticulitis are frequently seen in middle-aged and elderly populations. Acute gastritis, cardiac emergencies, and some metabolic emergencies can also present as acute abdominal pain in this age group [16-19]. Jameel et al. (2023) reported a similar trend, with a mean patient age of 41.4 ± 16.5 years [20]. This trend may be attributed to the increased risk of gastrointestinal and renal conditions among middle-aged adults influenced by lifestyle and dietary factors. Furthermore, the study found a higher prevalence of males n = 103 (58.52%) compared to females n = 73 (41.48%). This gender disparity in acute abdominal conditions might be due to the higher likelihood of males presenting with such conditions, potentially due to factors like higher rates of alcohol consumption, smoking, and occupational hazards. This observation is consistent with the findings of Jameel et al. (2023), who reported that there were 82 (53.2%) males and 72 (46.8%) females in their study [20].

Provisional diagnoses

In the present study, acute appendicitis was the most common diagnosis, accounting for n = 41 (23.29%) of cases. This finding differs from Bhagat et al. (2019), who reported acute cholecystitis as the most common etiology of acute abdominal pain, constituting 35.8% of cases [4]. Bhagat et al. (2019) also identified renal stones (30.5%), acute appendicitis (17.5%), and intestinal obstruction (16.67%) as common causes. The discrepancy between these studies could be attributed to variations in the clinical profiles and populations across different centers [4]. The second most common diagnosis in the present study was renal calculi, representing n = 33 (18.75%), bowel obstruction n = 12 (6.81%), and pancreatitis n = 12 (6.81%). This highlights the rising incidence of renal calculi, which may be influenced by dietary habits and climate change. These conditions, often associated with significant morbidity, necessitate rapid diagnosis and treatment. The role of alcohol consumption and gallstones in the etiology of pancreatitis has been well-documented, aligning with the findings from Bhagat et al. (2019) [4]. Similarly, Thakur et al. found acute appendicitis to be the most common diagnosis, followed by cholecystitis and renal colic, reflecting the heterogeneity in clinical profiles across various centers. Overall, these comparisons underscore the variability in the prevalence of acute abdominal conditions across different studies, potentially due to differences in study populations, regional factors, and clinical settings [21].

Disposition of patients

In the present study, the majority n = 98 (55.68%) of patients required conservative management. This is in contrast to the findings of Coccolini et al. (2021), who reported a pooled prevalence of surgical acute abdomen at 41.3% (95% confidence interval (CI): 39.7%-42.9%), found that most patients (73.8%) were male, with the main causes of surgical acute abdomen being acute appendicitis (42.1%; 95% CI: 40.0%-44.2%), bowel obstruction (35.9%; 95% CI: 33.8%-37.9%), and perforated peptic ulcers (4.5%; 95% CI: 3.5%-5.5%). In contrast, the present study's leading causes were acute appendicitis n =41 (24.43%) and renal calculi n = 33 (18.75%). Additionally, Coccolini et al. (2021) noted that complications occurred in 19.8% of cases (95% CI: 18.1%-21.5%), with a mortality rate of 5.1% (95% CI: 4.2%-6.0%). The present study did not specify complication and mortality rates, but the higher rate of surgical intervention n = 78 (44.31%) suggests a potentially more urgent or severe clinical presentation among the study population [22]. Overall, both studies emphasize the significant proportion of patients requiring surgical intervention for acute abdominal conditions. The remaining n = 78 (44.31%) of patients were managed medically, highlighting the importance of non-surgical interventions in acute care. This distribution is similar to that reported by Søreide et al. (2015), who emphasized the role of conservative management in conditions like gastritis and pancreatitis [23].

Pain assessment

Pre-treatment pain scores: The overall pain score before treatment had a mean of 6.27, indicating moderate to severe pain levels. This is consistent with findings by Cepeda et al. (2018), who reported similar baseline pain levels in acute abdomen patients [24]. Post-treatment pain scores: Significant reductions in pain scores post-treatment, with the mean VAS decreasing from 6.22 to 2.33 and the mean NRS dropping from 6.05 to 2.10, demonstrate effective pain management. A paired t-test was applied for the statistical analysis, and a p-value of <0.001 was obtained, indicating statistical significance, corroborating findings by Chumpitazi et al. (2015) [25] and Green et al. (2018) [26], who also reported significant pain reduction with appropriate analgesic protocols.

This study highlights the critical importance of prompt and accurate diagnosis, effective pain management, and appropriate disposition in patients presenting with acute abdomen. The findings underscore the need for comprehensive care strategies that address both the primary condition and associated comorbidities to optimize patient outcomes.

Limitation

This study has several limitations that need to be acknowledged. Firstly, the sample size was relatively small and confined to a single tertiary care hospital, which may limit the generalizability of the findings to other settings or populations. Secondly, the study was conducted over a specific period, which may not account for seasonal variations in the incidence of acute abdominal conditions. Thirdly, the exclusion of certain patient groups, such as those with traumatic acute abdomen and pregnant patients, may have introduced selection bias. Additionally, the study relied on the accuracy and completeness of medical records and patient self-reports, which could be subject to information bias. Lastly, the lack of long-term follow-up data means that the study could not assess the outcomes of the patients beyond their initial treatment and discharge from the emergency department. Future studies with larger, multi-center cohorts and extended follow-up periods are recommended to address these limitations and provide more robust data.

## Conclusions

This study highlights the significant burden of gastrointestinal and renal conditions, with acute appendicitis and urolithiasis being the most common diagnoses in patients presenting with acute abdomen. The high rate of surgical interventions underscores the urgent nature of many of these conditions. The significant reduction in pain scores post-treatment demonstrates the effectiveness of the pain management strategies employed. These findings emphasize the importance of prompt and accurate diagnosis, effective pain management, and appropriate patient disposition in the management of acute abdomen. Comprehensive care strategies are essential for optimizing patient outcomes, and future research with larger sample sizes and multi-center participation is recommended to validate these findings and enhance management protocols.

## Appendices

Table 7 represents the proforma used for the patients presented to our emergency department with acute abdominal pain.

**Table 7:** Proforma.

Case no	
Name	
Age	
Gender	
Address	
Rural/urban	
Occupation	
Annual income	
Phone	
OP/IP no	
History	
Duration of symptoms	
Vomiting/LOC/visual disturbance	
H/o trauma, similar episodes in past	
Addictions	
General physical examination	1. General examination: Temp, JVP, pulse, clubbing, edema, pallor, lymph node, cyanosis. 2. Vital signs: pulse (beats per minute), blood pressure (mm of Hg)
Systematic examination	Respiratory system, cardiovascular system, central nervous system and per abdomen: inspection: auscultation; percussion; palpation; site, onset: sudden or gradual, character: localized or diffuse, dull or sharp, associated symptoms: jaundice, anorexia, nausea and/or vomiting, diarrhea/constipation, pyrosis and/or singultus, genitourinary symptoms–dysuria, hematuria-time course: Is the pain continuous or intermittent in nature, exacerbating/relieving factors: Are there palliating or provoking factors–breathing, coughing, food intake, defecation, vomiting; severity: from 1-10 on the visual analog scale (VAS) and numerical rating score (NRS), digital rectal examination
Imaging	Ultrasonography (abdomen + pelvis)
Pain score before treatment (visual analog score)	
Pain score after treatment (visual analog score)	
Pain score before treatment (numerical rating score)	
Pain score after treatment (numerical rating score)	
Disposition	Shifted to operation theater or shifted to ward
